# Supplementary material for: Secondary structural ensembles of the SARS-CoV-2 RNA genome in infected cells
Source: Nat Commun. 2022 Mar 2;13:1128. doi: 10.1038/s41467-022-28603-2 (PMC8891300; doi:10.1038/s41467-022-28603-2)
Supplement: Supplementary file 1 — Supplementary Information [file 41467_2022_28603_MOESM1_ESM.pdf]

Supplementary Information for

"Secondary structural ensembles of the SARS-CoV-2 RNA genome in infected cells"

Contents:

Supplementary Figures 1 – 11

(Supplementary Data 1 – 8 are provided as separate files.)

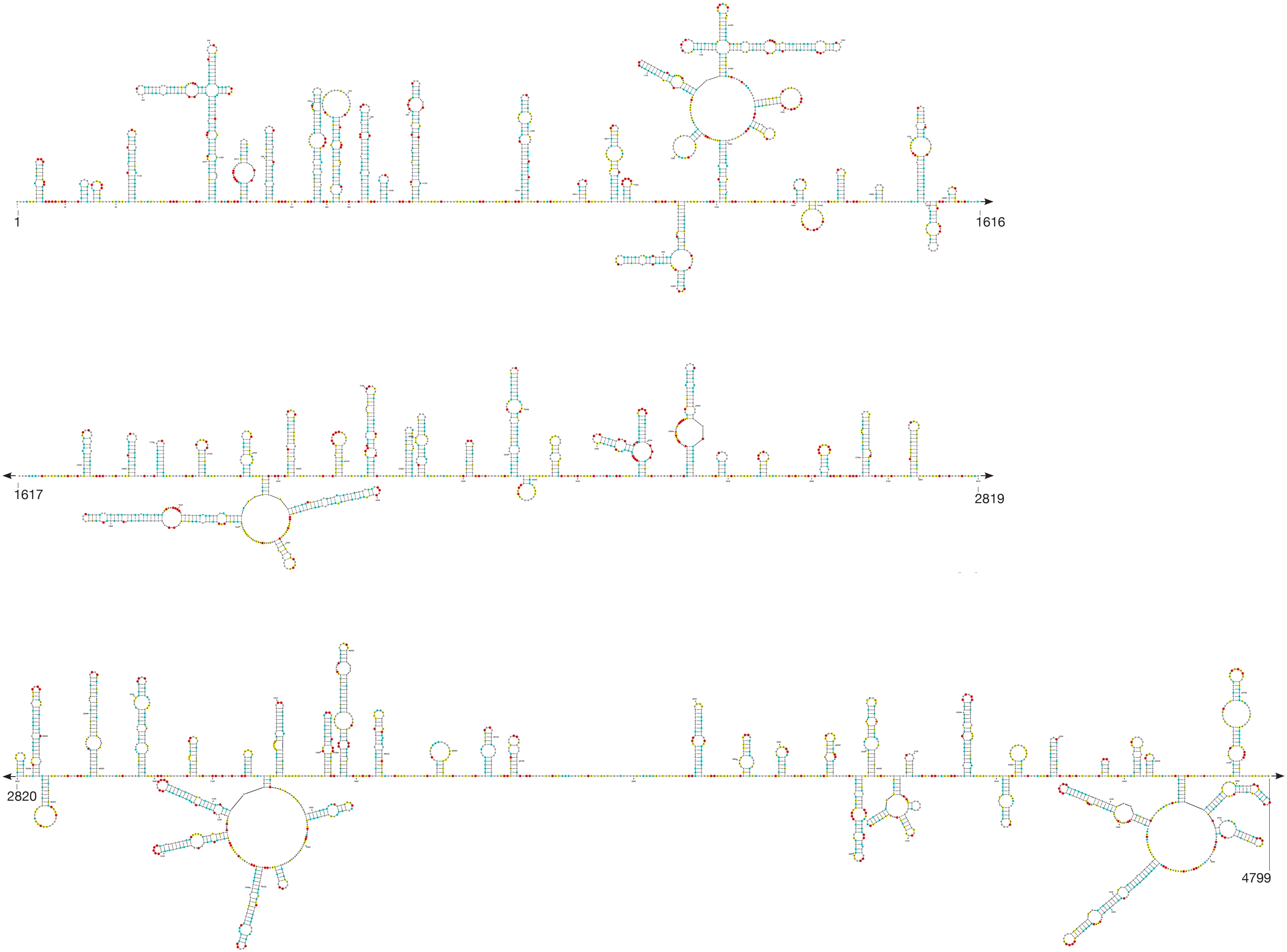
1
1616
1617
2819
2820
4799

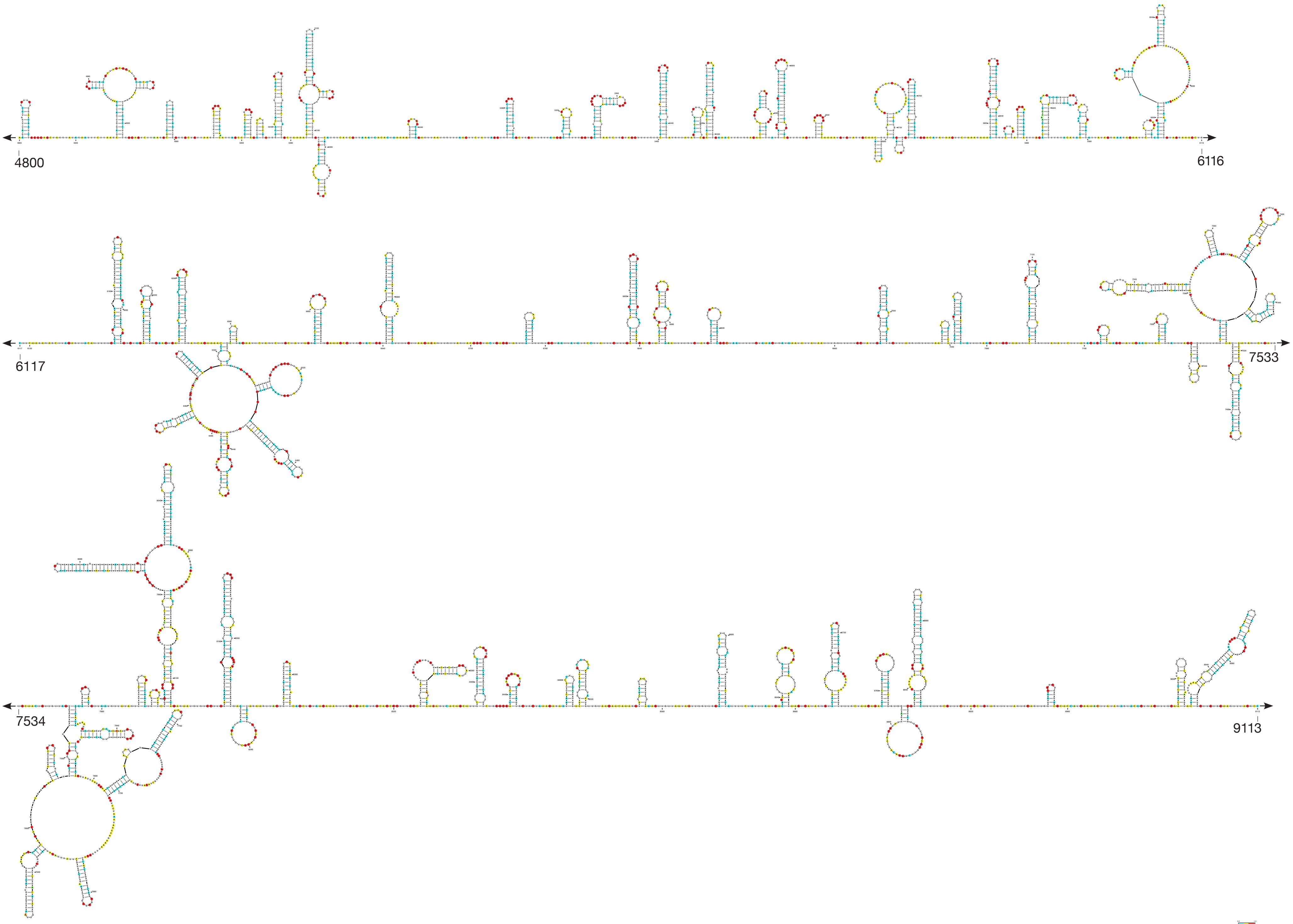
4800
6116
6117
7533
7534
9113

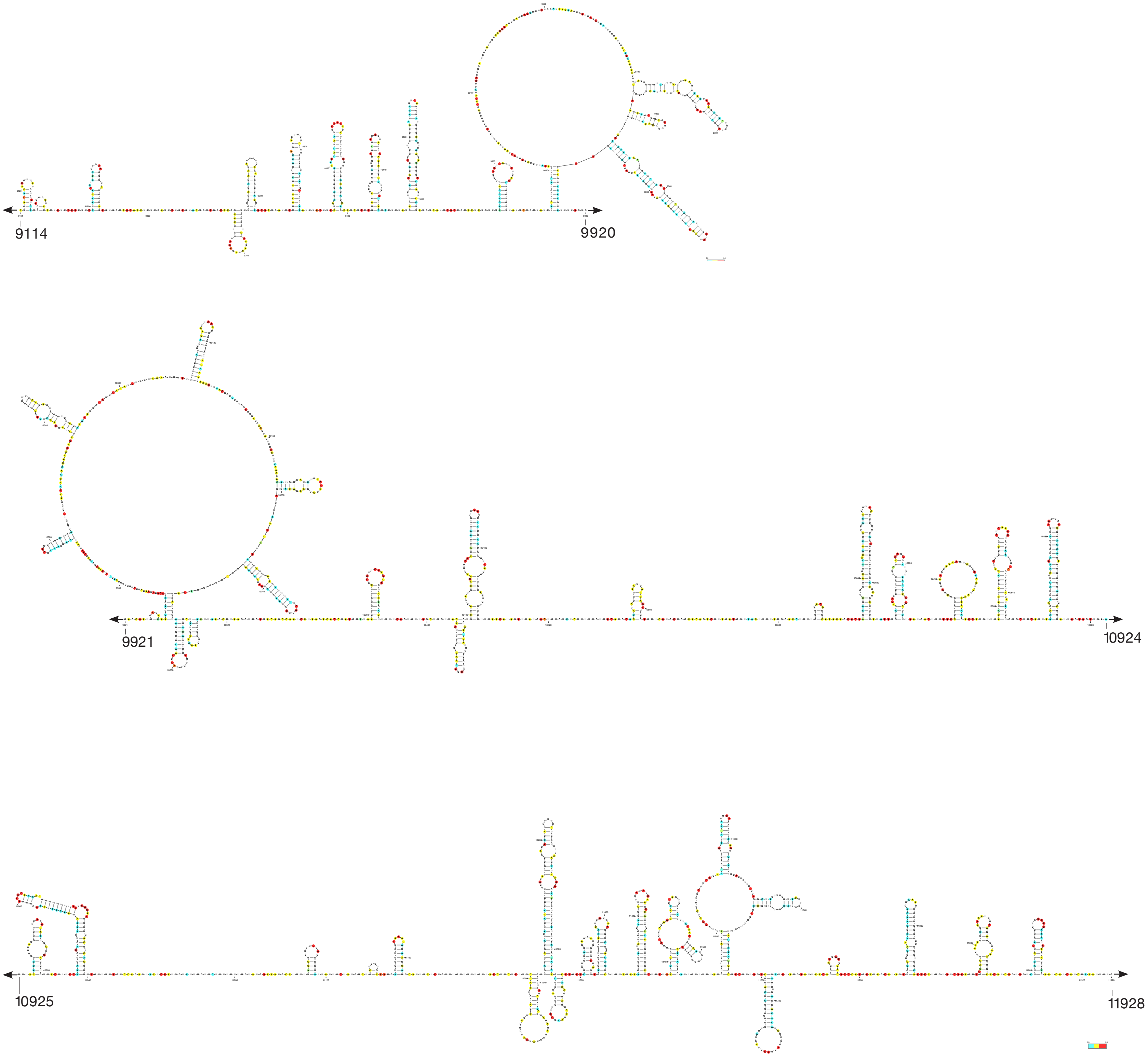
9114
9920
9921
10924
10925
11928

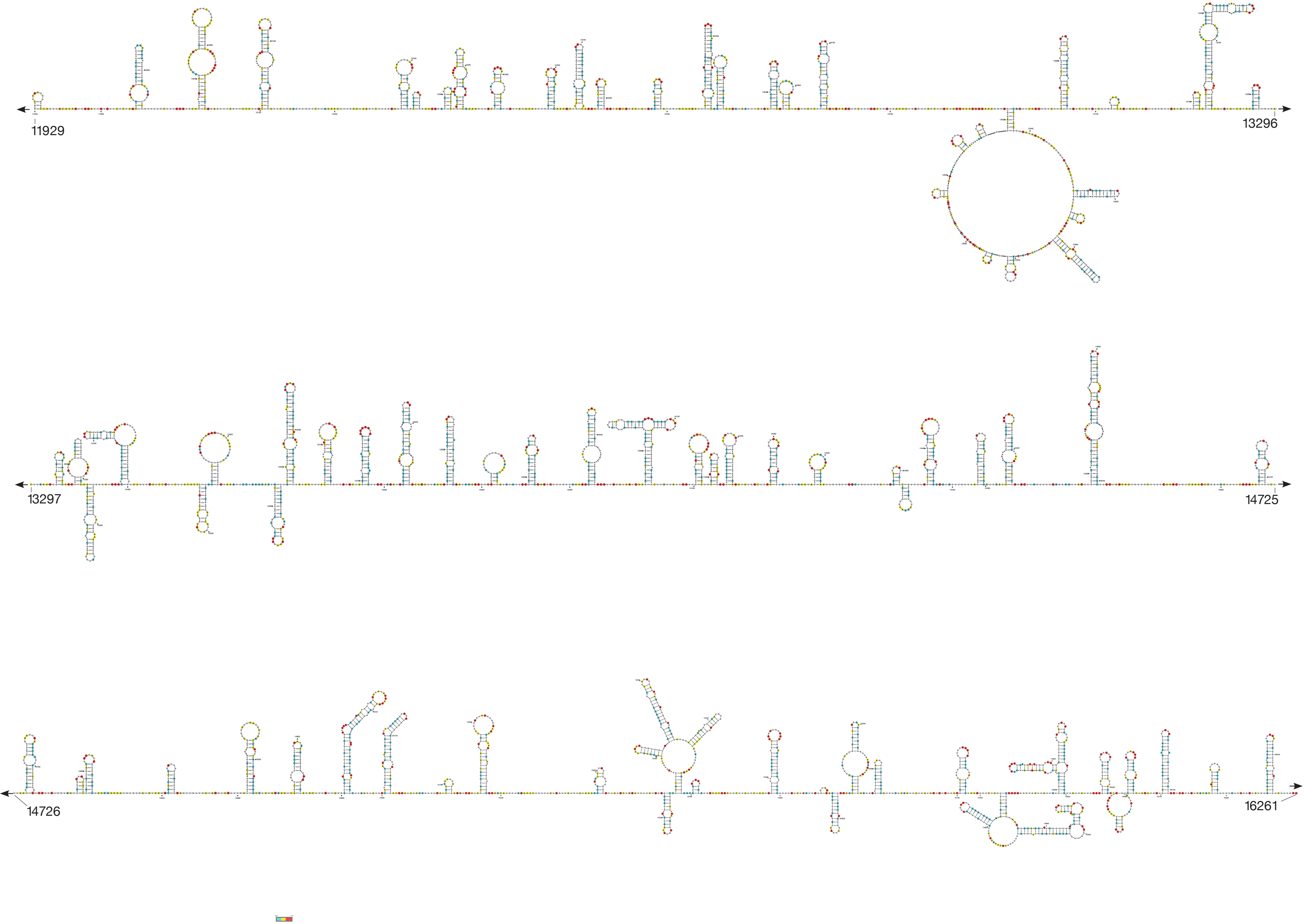
11929
13296
13297
14725
14726
16261

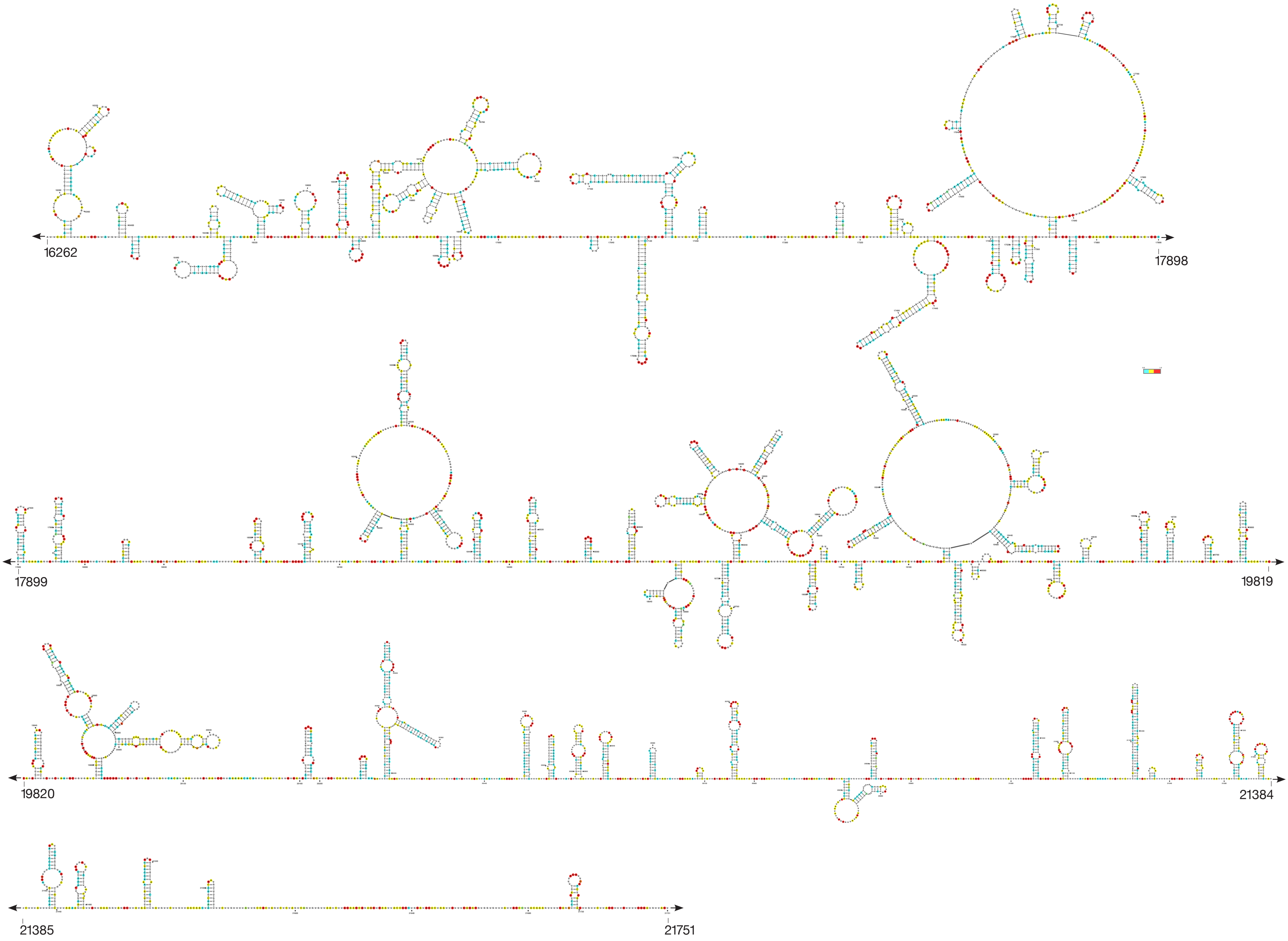
16262
17898
17899
19819
19820
21384
21385
21751

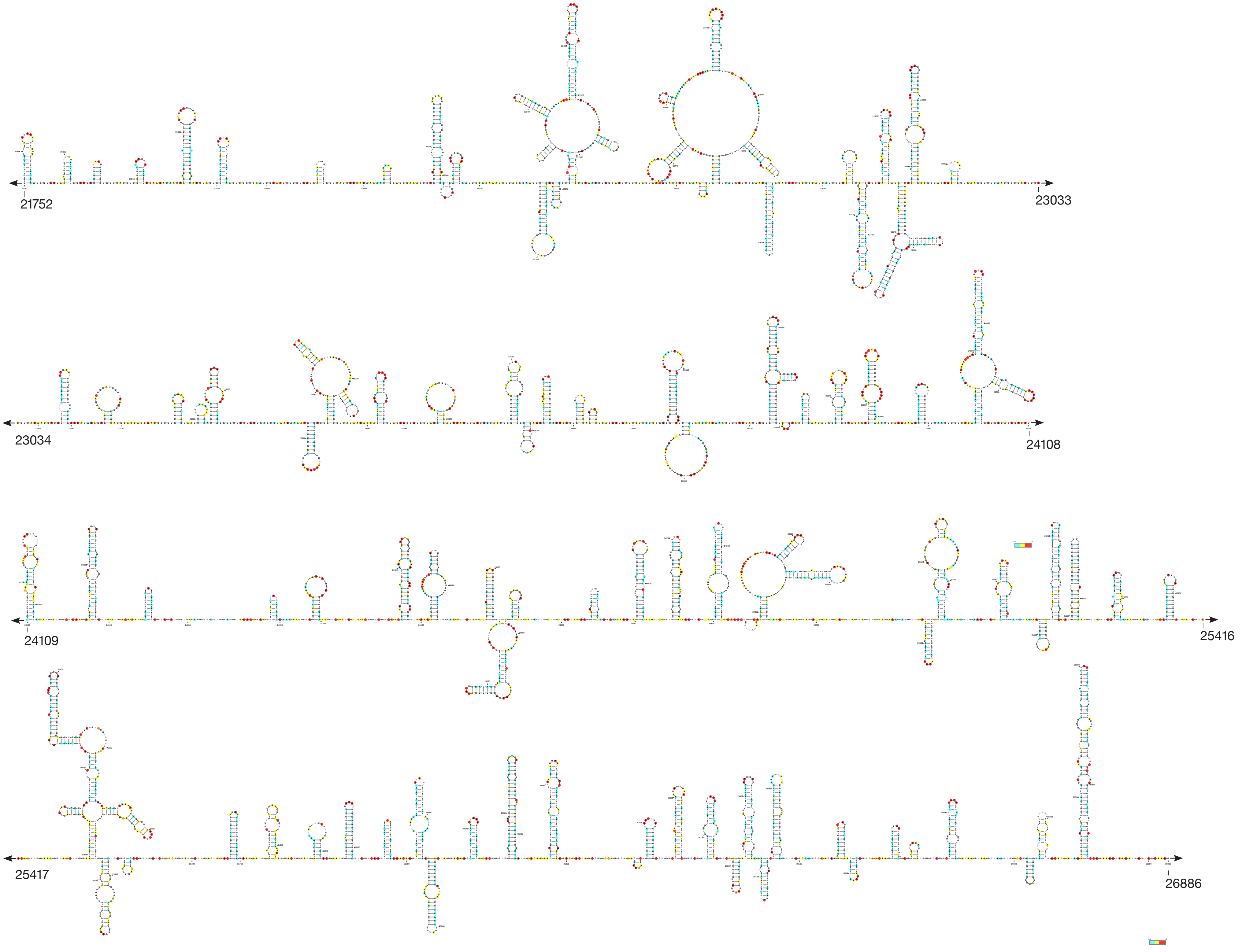
21752
23033
23034
24108
24109
25416
25417
26886

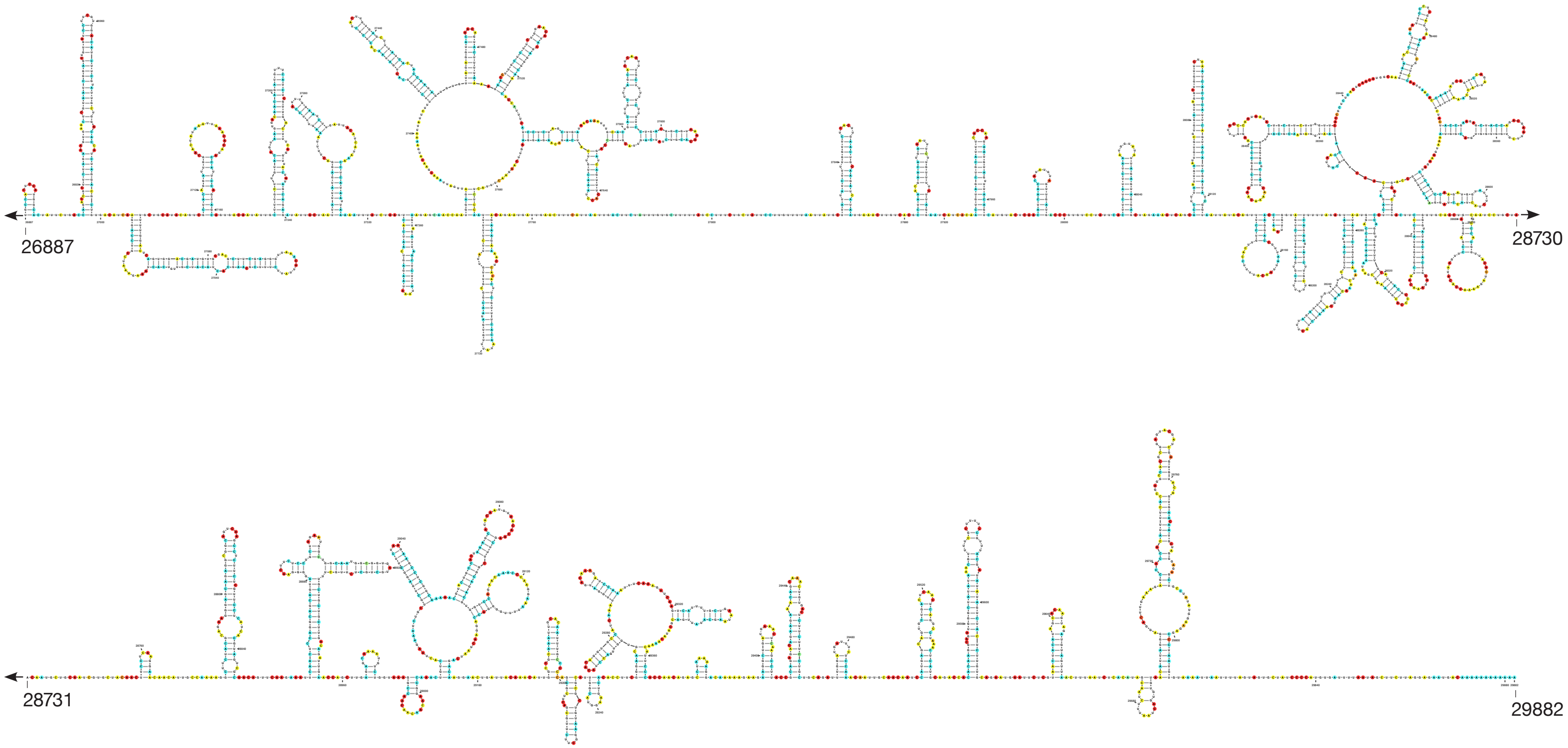

Supplementary Figure 1: Data-derived secondary structure of the full SARS-CoV-2 genome in Vero cells. The predicted secondary structure of the full genome of severe acute respiratory syndrome coronavirus 2 (SARS-CoV-2) based on population average reactivities from dimethyl sulfate mutational profiling with sequencing (DMS-MaPseq). Bases are colored according to their normalized DMS reactivities.

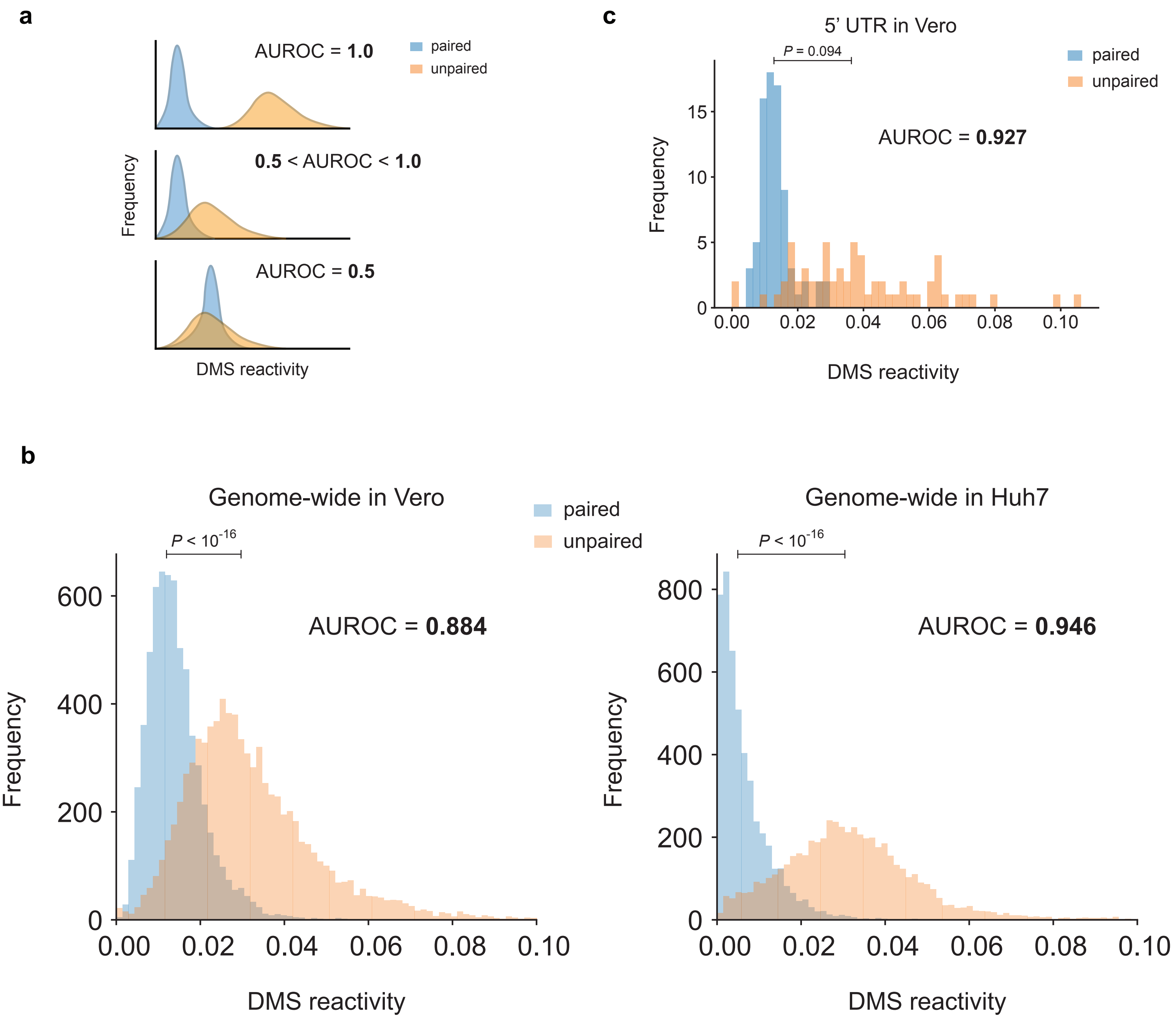

Supplementary Figure 2: Distributions of DMS reactivities on paired and unpaired bases, and relationship to AUROC.

(a) Schematic showing the relationship between dimethyl sulfate (DMS) reactivity distributions and area under the receiver operating characteristic curve (AUROC). When the DMS reactivity of every paired nucleotide is lower than that of every unpaired nucleotide, AUROC = 1.0 (left). If the distributions overlap but unpaired nucleotides have higher median DMS reactivity, then 0.5 < AUROC < 1.0 (middle). If the median DMS reactivities of paired and unpaired bases are equal, then AUROC = 0.5 (right).

(b) Histograms of DMS reactivities on paired and unpaired adenines (As) and cytosines (Cs) across the entire SARS-CoV-2 genome in Vero cells (left) and Huh7 cells (right). AUROC values are shown. For clarity, histograms are truncated at 0.10, which captures 99.6% and 99.4% of all Vero and Huh7 data, respectively. The bar indicates the difference in median reactivities of paired and unpaired nucleotides. $P < 10^{-16}$ for difference in medians (two-sided Mann-Whitney *U* test).

(c) Histogram of DMS reactivities on paired and unpaired As and Cs across the 5' untranslated region (UTR) stem loops 1 – 5 (coordinates 1 – 294) in Vero cells. $P = 0.094$ (two-sided Mann-Whitney *U* test). Source data are provided as a Source Data file.

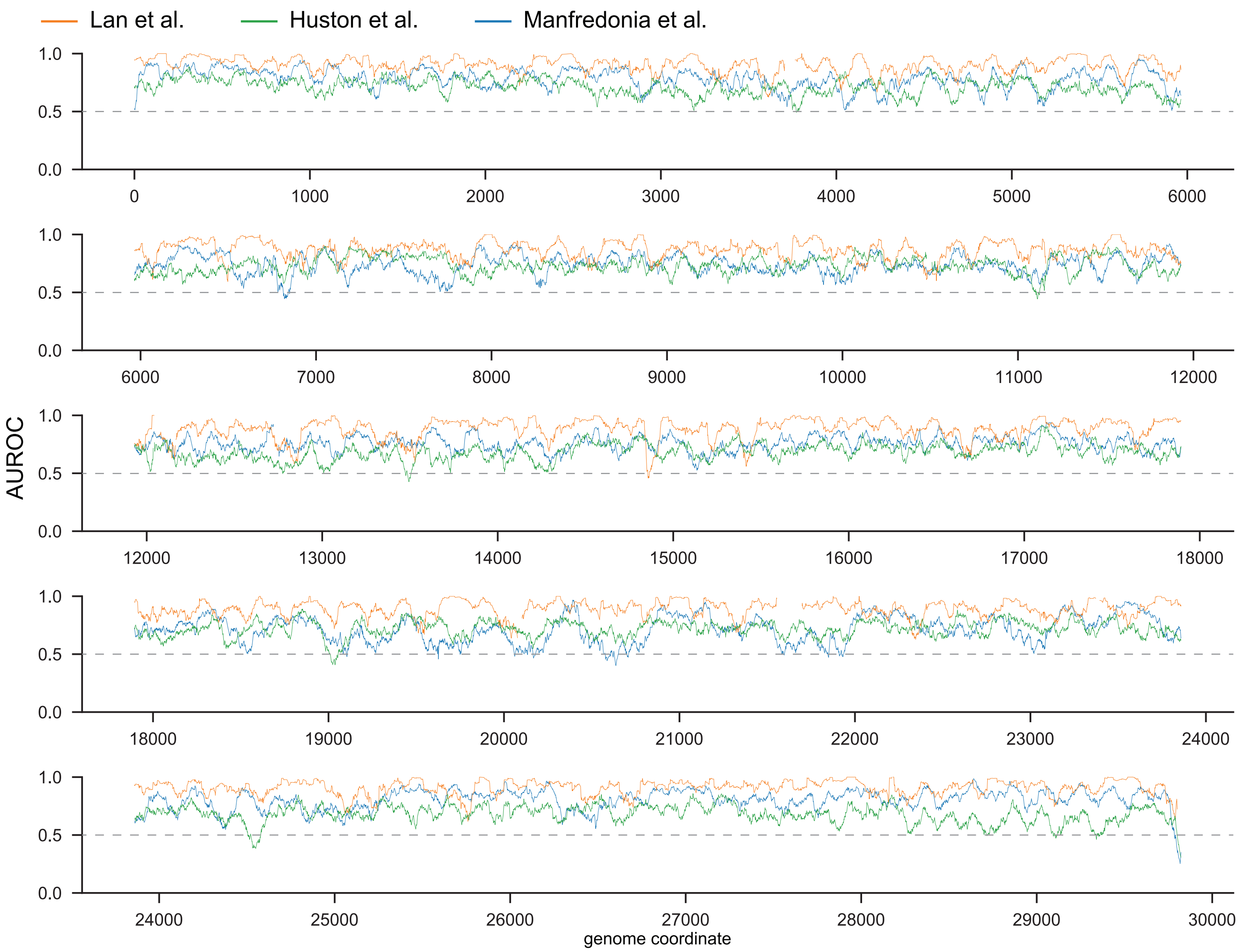

Supplementary Figure 3: Local data-structure agreement (AUROC) from this study, Huston et al., and Manfredonia et al.
The area under the receiver operating characteristic curve (AUROC) between the predicted population average structure and the DMS/SHAPE reactivities was computed using a sliding window of 80 nt, incremented by 1 nt, for our model in Vero cells and the Vero cell models of Huston et al. and Manfredonia et al. Windows with fewer than 5 paired or 5 unpaired nucleotides with DMS/SHAPE reactivities were left blank. The dashed gray line at AUROC = 0.5 indicates the expected AUROC if the DMS/SHAPE reactivities were randomly shuffled. Source data are provided as a Source Data file.

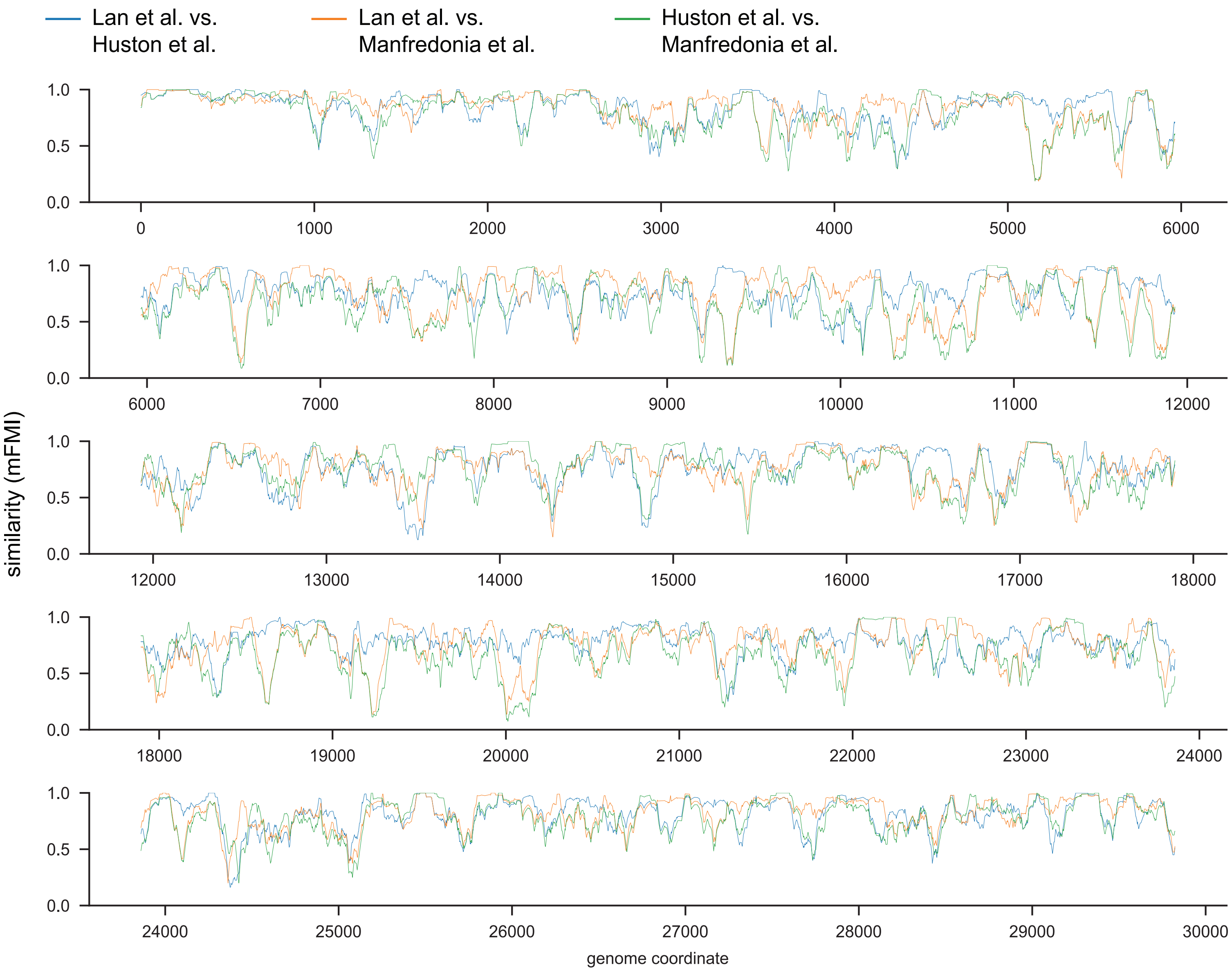

Supplementary Figure 4: Genome-wide pairwise similarity of population average models from this study, Huston et al., and Manfredonia et al.
The structural similarity, measured by the modified Fowlkes-Mallows index (mFMI), for each pair of genome-wide secondary structure models of SARS-CoV-2 based on population average DMS/SHAPE reactivities. The three models used in this figure are from this work, Huston et al., and Manfredonia et al. (all from infected Vero cells). The mFMI was computed using a sliding window of 80 nt, incremented by 1 nt. Source data are provided as a Source Data file.

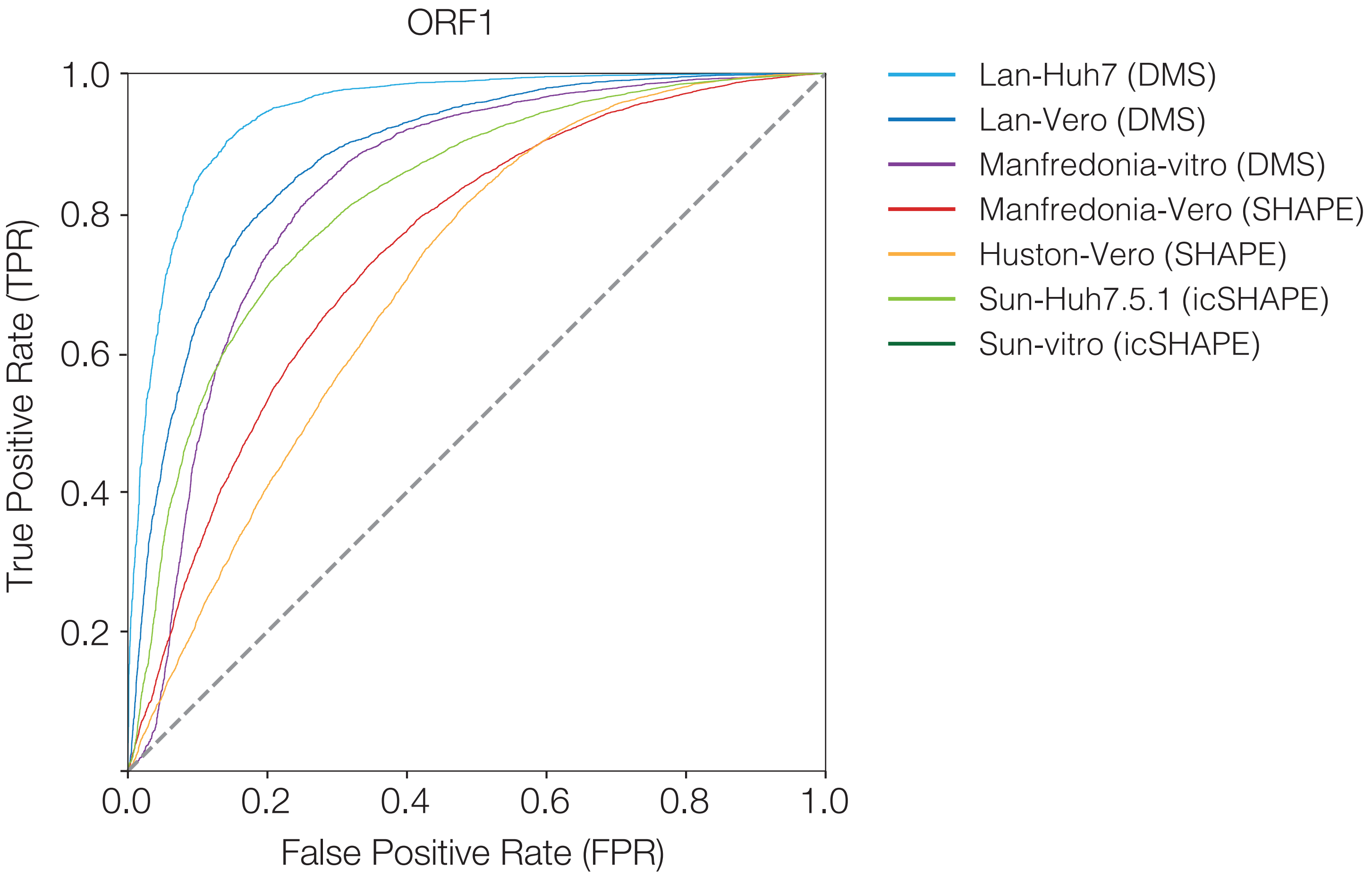

Supplementary Figure 5: Receiver operating characteristic (ROC) curves for population average structures DMS/SHAPE/icSHAPE reactivities over only ORF1. To eliminate any possible effects of subgenomic RNAs on the receiver operating characteristic (ROC), the ROC curve over open reading frame 1 (ORF1, present only in genomic RNA; coordinates 266 – 21555) was computed for each DMS/SHAPE dataset and corresponding secondary structure model. For each population average structure, the first author, cell type (or "vitro" for *in vitro*), and chemical probe family are given. Source data are provided as a Source Data file.

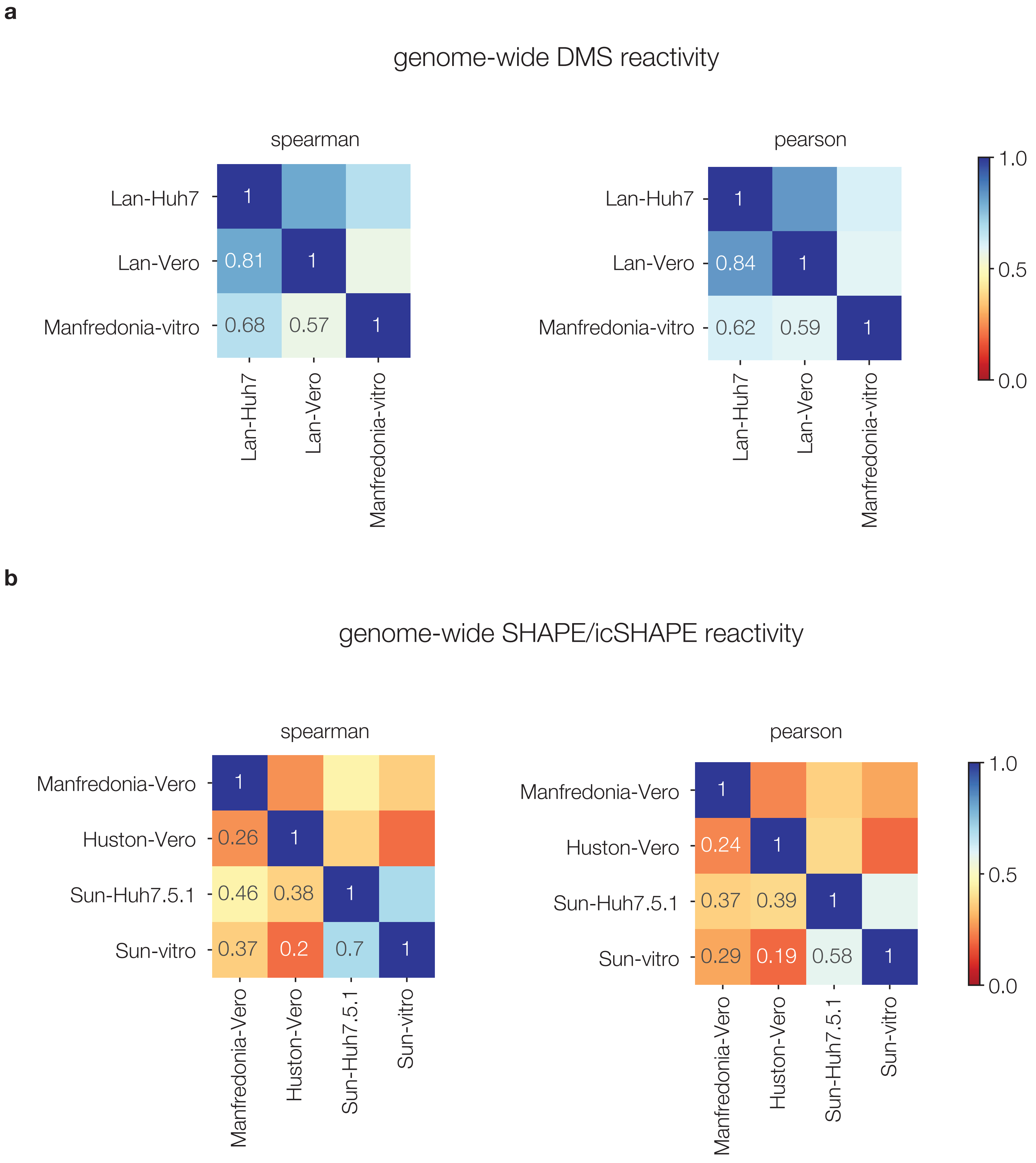

Supplementary Figure 6: Comparison of genome-wide chemical probing datasets. **(a)** Comparison of different DMS reactivity datasets (our datasets in Vero and Huh7 cells, and Manfredonia et al. *in vitro*) using Spearman (left) and Pearson (right) correlations. **(b)** Comparison of different SHAPE reactivity datasets (Manfredonia et al. and Huston et al. in Vero cells) and icSHAPE datasets (Sun et al. in Huh7.5.1 cells and *in vitro*) using Spearman (left) and Pearson (right) correlations. Source data are provided as a Source Data file.

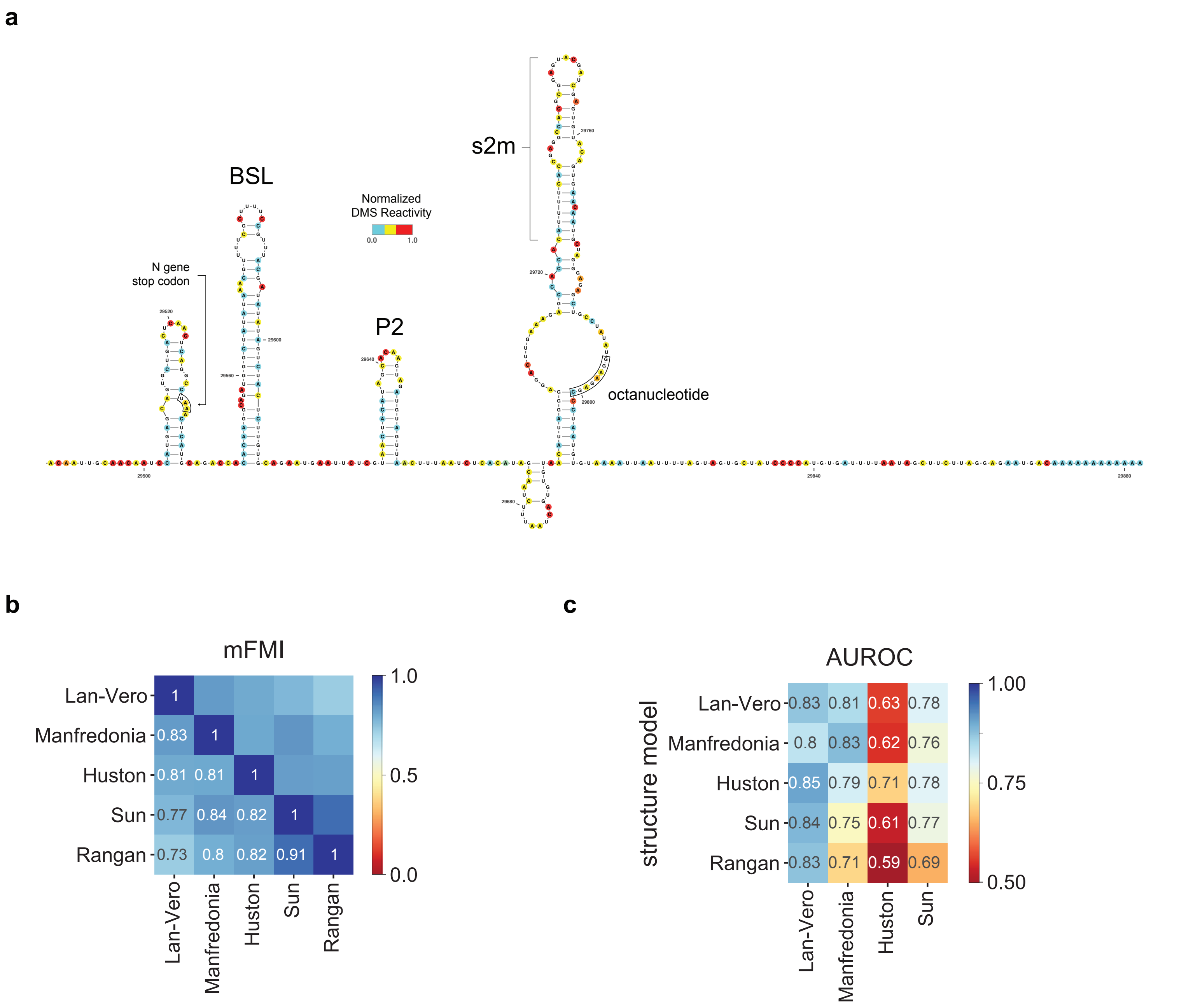

Supplementary Figure 7: Model of the 3' untranslated region (UTR).
(a) Our population average structure model of the 3' UTR from Vero cells. Known structural features including the bulged stem loop (BSL), the P2 stem loop, and the stem-loop 2 motif (s2m) are labeled. Nucleotides are colored by normalized DMS reactivities. Loss of DMS signal at the poly(A) tail is consistent with the known behavior of the TGIRT enzyme.
(b) Similarity (measured as modified Fowlkes-Mallows index, mFMI) of each pair of structural models of the 3' UTR (from this work, Manfredonia et al., Huston et al., Sun et al., and Rangan et al.), over coordinates 29543 – 29870.
(c) Cross-agreement (measured as area under the receiver operating characteristic curve, AUROC) between each structure model (from this work, Manfredonia et al., Huston et al., Sun et al., and Rangan et al.) and each DMS/SHAPE/icSHAPE reactivity dataset (from this work, Manfredonia et al., Huston et al., and Sun et al.) of the 3' UTR over coordinates 29543 – 29870. Source data are provided as a Source Data file.

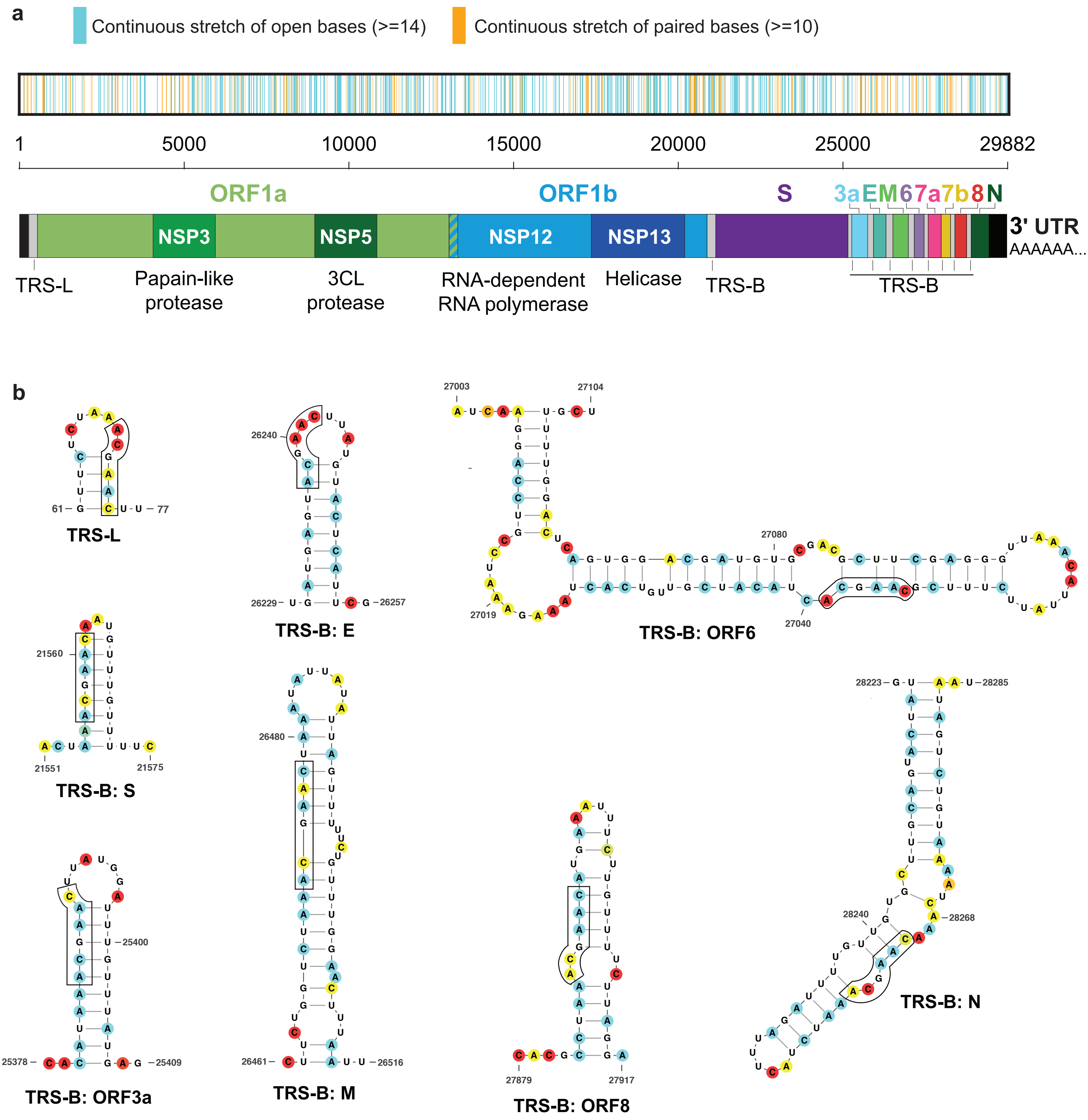

Supplementary Figure 8: Structured and unstructured regions in the SARS-CoV-2 genome.

(a) Locations of highly structured and unstructured regions in the SARS-CoV-2 genome model from Vero cells. Highly structured regions are defined are stretches of at least 10 consecutive paired bases; unstructured regions shown are stretches of at least 14 consecutive unpaired bases. The thickness of each bar is proportional to the number of consecutive paired (blue) or unpaired (orange) bases. The data is plotted over a schematic of the genome, highlighting the organization of open reading frames (ORFs) and the transcription-regulating sequences (TRS).

(b) In-cell model of each of the eight transcription-regulating sequences (TRSs) predicted to lie within a stem loop. The core sequence (CS) of each TRS is outlined in black. Models are arranged in genomic order from top-to-bottom, left-to-right. Source data are provided as a Source Data file.

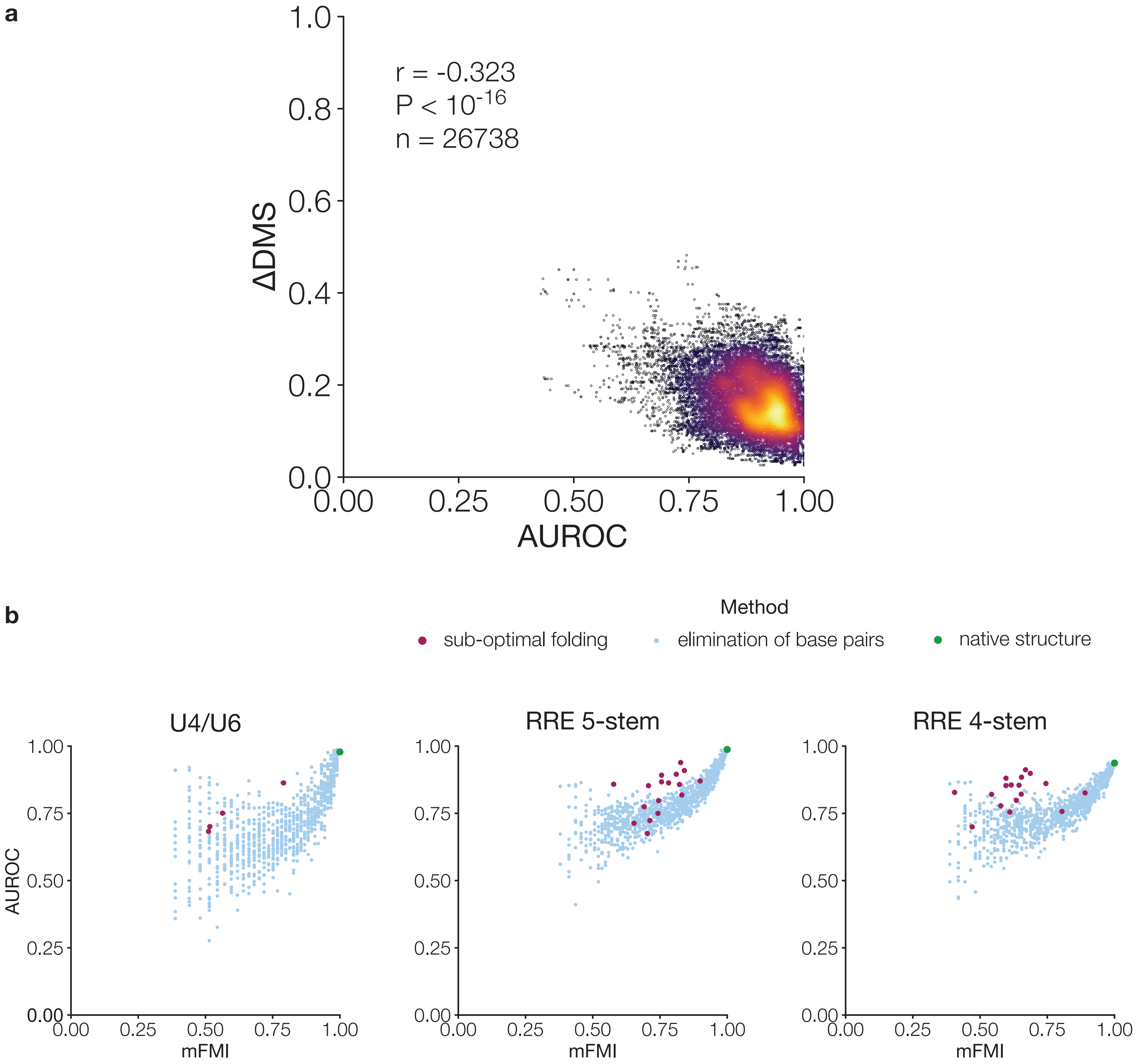

Supplementary Figure 9: Agreement between the reactivity data and predicted structure is related positively with similarity to the ground truth structure and negatively with structural heterogeneity.

(a) Scatterplot heatmap of the local agreement between the predicted structure and the DMS reactivity data versus the amount of local structural heterogeneity. Each of the n=26,738 points represents one 80 nt window from our Vero model of the SARS-CoV-2 genome. Data-structure agreement (measured as area under the receiver operating characteristic curve, AUROC) and structural heterogeneity (measured as the average difference between DMS reactivities between the two clusters, if present) were computed for each 80 nt window in increments of 1 nt. The Pearson correlation (r) is shown. $P < 10^{-16}$, two-tailed beta distribution over the interval [-1, 1] with parameters $\alpha=n/2-1$, $\beta=n/2-1$.

(b) Scatterplot of agreement with DMS reactivities (AUROC) versus similarity to ground truth structures (mFMI) for 1) the RNA sequence folded without DMS constraints into sub-optimal structures and 2) ground truth structures from which base pairs were eliminated. The location of the ground truth structure is also shown on each plot. Source data are provided as a Source Data file.

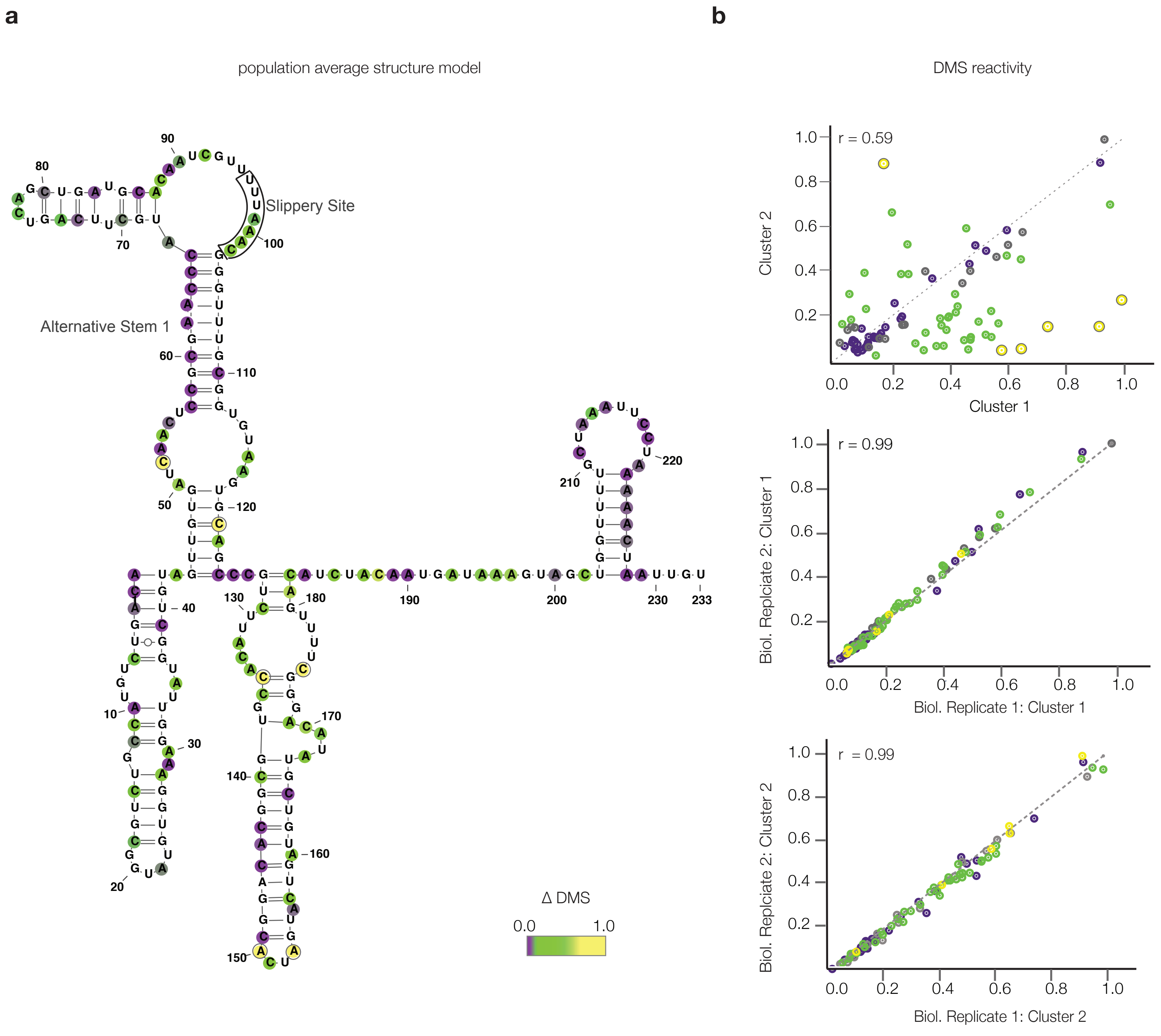

Supplementary Figure 10: RNA structure dynamics of the frameshifting stimulation element (FSE).

(a) Population average structure of the FSE from Vero cells. Alternative stem 1 and slippery site sequences are marked. Nucleotides are colored by change in DMS reactivity (ΔDMS) between clusters 1 and 2. The same colors are used on scatterplots in (b) showing the comparison of DMS signal between clusters.

(b) Scatterplots of DMS reactivities comparing cluster 1 and 2 within a biological replicate (top) and comparing biological replicates for each cluster (middle, bottom). The dotted line is the identity line; r is Pearson's coefficient. The ΔDMS is the normalized distance of each point (i.e. nucleotide) to the identity line. Source data are provided as a Source Data file.

a

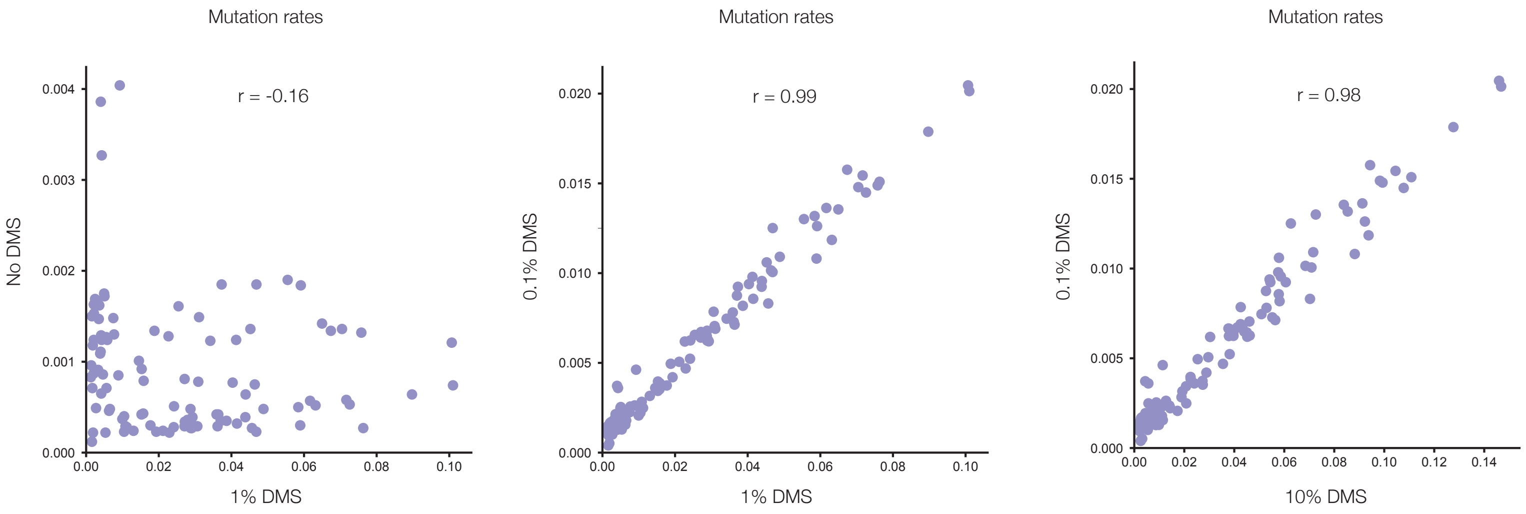

b

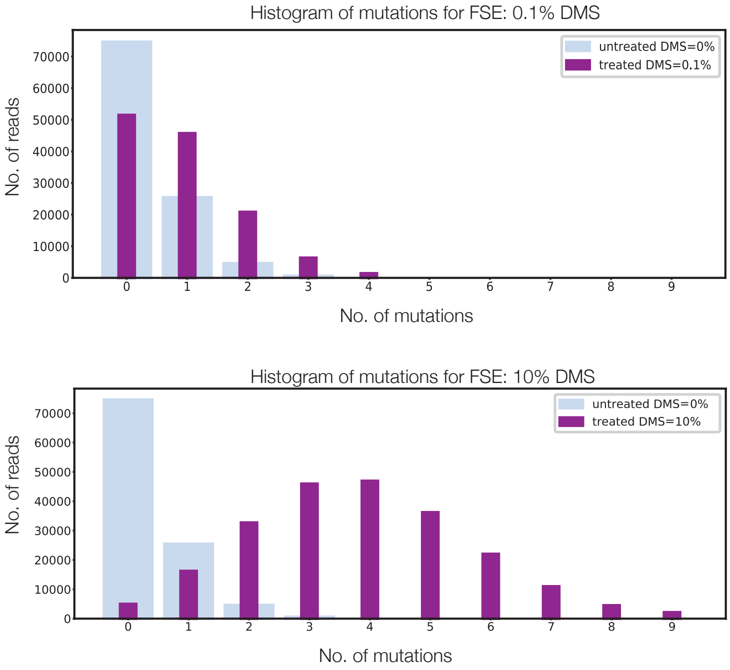

c

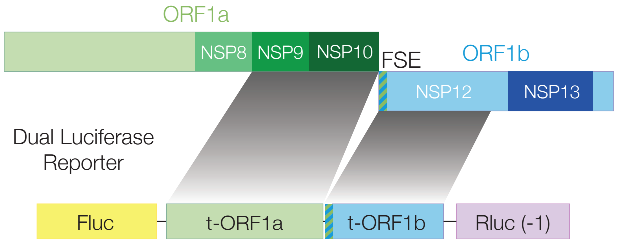

vs.	3kb FSE without luciferase reporter in cells	viral FSE in Huh-7 cells
3kb FSE with luciferase reporter in cells	r = 0.99	r = 0.82

Supplementary Figure 11: Robustness of DMS reactivities to number of modifications per molecule and addition of luciferase sequences.
(a) Scatterplots comparing mutation rates of each nucleotide in an RNA segment containing the SARS-CoV-2 frameshifting stimulation element (FSE) at different DMS concentrations: no DMS vs. 1% DMS (left), 0.1% DMS vs 1% DMS (middle), and 0.1% DMS vs. 10% DMS (right). Pearson correlations (r) are shown.
(b) Histograms of number of mutations per read at 0.1% DMS (top, purple) and 10% DMS (bottom, purple); mutations without DMS are shown in lavender.
(c) Comparison of unfiltered DMS reactivities over the FSE in the 2924 nt (~3 kb) construct with added firefly luciferase (Fluc) and Renilla luciferase (Rluc) reporters (left) to the 3 kb construct without reporters (top) and the full genome in Huh7 cells (right). Pearson correlations (r) are shown. The 3 kb segment contains the FSE and includes upstream and downstream portions of truncated open reading frame 1a (t-ORF1a) and 1b (t-ORF1b). Source data are provided as a Source Data file.
