# Supplementary material for: Secondary structural ensembles of the SARS-CoV-2 RNA genome in infected cells
Source: Nat Commun. 2022 Mar 2;13:1128. doi: 10.1038/s41467-022-28603-2 (PMC8891300; doi:10.1038/s41467-022-28603-2)
Supplement: Supplementary file 3 — Description of Additional Supplementary Files [file 41467_2022_28603_MOESM3_ESM.pdf]

Description of Additional Supplementary Files

File Name: Supplementary Data 1
Description: List of significantly (E-value < 0.05) covarying base pairs within the Vero-based model of the SARS-CoV-2 genome. The distance between the bases and the first and last bases within the structural element containing the base pair are given. The remaining columns are outputs from R-scape describing whether the two covarying bases are paired in the genome-wide Vero structure model, the R-scape score, the E-value, the number of substitutions found in the dataset, and the R-scape power.

File Name: Supplementary Data 2
Description: List of each secondary structural element that contains at least one covarying base pair with E-value < 0.05 in the Vero-based model of the SARS-CoV-2 genome. For each element, the positions of the first and last bases, the number of significantly covarying pairs, and a list of those pairs is given.

File Name: Supplementary Data 3
Description: List of all regions of the Huh7 genome where the DMS reactivities formed at least two clusters (i.e. alternative structures) according to our criteria (see Methods). For each region, the start and end positions of the region, the number of clusters formed by the region (up to 3), the % abundance of each cluster (comma-separated list), and similarity (R^2) between all pairs of clusters (comma-separated list; for regions with 3 clusters, order is 1 vs 2, 1 vs 3, 2 vs 3).

File Name: Supplementary Data 4
Description: For each base within a Huh7 region forming at least two clusters (see Supplementary Data 3), the number of clusters and the filtered DMS reactivity of the base in each cluster is given. DMS reactivities of guanines and uracils are set to zero.

File Name: Supplementary Data 5
Description: List of primers used in this study.

File Name: Supplementary Data 6
Description: Genome-wide secondary structure model of SARS-CoV-2 base on DMS reactivities in Vero cells, in connectivity table (CT) format.

File Name: Supplementary Data 7
Description: Genome-wide secondary structure model of SARS-CoV-2 base on DMS reactivities in Huh7 cells, in connectivity table (CT) format.

File Name: Supplementary Data 8
Description: Population average DMS reactivities for the datasets from Huh7 cells (filtered) and Vero cells (filtered), as well as from each replicate in Vero cells (unfiltered).
