# Supplementary material for: Secondary structural ensembles of the SARS-CoV-2 RNA genome in infected cells
Source: Nat Commun. 2022 Mar 2;13:1128. doi: 10.1038/s41467-022-28603-2 (PMC8891300; doi:10.1038/s41467-022-28603-2)
Supplement: Supplementary file 12 — Reporting Summary [file 41467_2022_28603_MOESM12_ESM.pdf]

nature research

Corresponding author(s): Silvi Rouskin

Last updated by author(s): Feb 12, 2020

Reporting Summary

Nature Research wishes to improve the reproducibility of the work that we publish. This form provides structure for consistency and transparency in reporting. For further information on Nature Research policies, see our Editorial Policies and the Editorial Policy Checklist.

Statistics

For all statistical analyses, confirm that the following items are present in the figure legend, table legend, main text, or Methods section.

n/a	Confirmed	
☐	☒	The exact sample size (*n*) for each experimental group/condition, given as a discrete number and unit of measurement
☐	☒	A statement on whether measurements were taken from distinct samples or whether the same sample was measured repeatedly
☐	☒	The statistical test(s) used AND whether they are one- or two-sided<br>*Only common tests should be described solely by name; describe more complex techniques in the Methods section.*
☒	☐	A description of all covariates tested
☐	☒	A description of any assumptions or corrections, such as tests of normality and adjustment for multiple comparisons
☐	☒	A full description of the statistical parameters including central tendency (e.g. means) or other basic estimates (e.g. regression coefficient) AND variation (e.g. standard deviation) or associated estimates of uncertainty (e.g. confidence intervals)
☐	☒	For null hypothesis testing, the test statistic (e.g. *F*, *t*, *r*) with confidence intervals, effect sizes, degrees of freedom and *P* value noted<br>*Give P values as exact values whenever suitable.*
☒	☐	For Bayesian analysis, information on the choice of priors and Markov chain Monte Carlo settings
☒	☐	For hierarchical and complex designs, identification of the appropriate level for tests and full reporting of outcomes
☐	☒	Estimates of effect sizes (e.g. Cohen's *d*, Pearson's *r*), indicating how they were calculated

Our web collection on statistics for biologists contains articles on many of the points above.

Software and code

Policy information about availability of computer code

Data collection	An iSeq 100 (Illumina), NextSeq 300 (Illumina), and Novaseq (Illumina) were used to collect sequencing data.
Data analysis	RNA structures were determined using the DREEM pipeline (see Methods). RNA structures were drawn using VARNA v3.93. Figures 1, 2, 3, S2, S3, S4, S5, S6, S7, S8, and S9 were generated using custom scripts available on GitHub (https://github.com/matthewfallan/SARS-CoV-2_genome_structure and https://github.com/matthewfallan/rouls).

For manuscripts utilizing custom algorithms or software that are central to the research but not yet described in published literature, software must be made available to editors and reviewers. We strongly encourage code deposition in a community repository (e.g. GitHub). See the Nature Research guidelines for submitting code & software for further information.

Data

Policy information about availability of data

All manuscripts must include a data availability statement. This statement should provide the following information, where applicable:

- Accession codes, unique identifiers, or web links for publicly available datasets
- A list of figures that have associated raw data
- A description of any restrictions on data availability

The short read sequencing data generated in this study have been deposited into NCBI Gene Expression Omnibus (GEO) under accession code GSE153851 [https://www.ncbi.nlm.nih.gov/geo/query/acc.cgi?acc=GSE153851].

Whole-genome secondary structure models of SARS-CoV-2 in Vero and Huh7 cells are provided in connectivity table format in Supplementary Data 6 and 7, respectively. Raw DMS reactivities for each biological replicate of Vero, Vero (aggregated replicates), and Huh7 are provided in Supplementary Data 8. A file of Source Data for all main and supplementary figures is provided with this paper.

Field-specific reporting

Please select the one below that is the best fit for your research. If you are not sure, read the appropriate sections before making your selection.

☒ Life sciences ☐ Behavioural & social sciences ☐ Ecological, evolutionary & environmental sciences

For a reference copy of the document with all sections, see nature.com/documents/nr-reporting-summary-flat.pdf

Life sciences study design

All studies must disclose on these points even when the disclosure is negative.

Sample size	No sample size calculations were performed because the number of points in the DMS-MaPseq data is fixed by the length of the SARS-CoV-2 genome, and consistency of replicates was first checked using a small number (n = 2 or 3, described in "Replication").
Data exclusions	Sequencing reads were excluded for failing to meet quality criteria, as described in Methods.
Replication	For genome-wide chemical probing experiments, two replicates were performed, and they were reproducible (R^2 = 0.874). For each dual luciferase assay, three biological replicates and two technical replicates for each biological replicate were performed, and there were no outliers.
Randomization	No randomization was necessary in this study because all specimens subject to different treatments came from the same source.
Blinding	Blinding was irrelevant to this study because all data were analyzed objectively using the same pipeline.

Reporting for specific materials, systems and methods

We require information from authors about some types of materials, experimental systems and methods used in many studies. Here, indicate whether each material, system or method listed is relevant to your study. If you are not sure if a list item applies to your research, read the appropriate section before selecting a response.

Materials & experimental systems

n/a	Involved in the study	
☒	☐	Antibodies
☐	☒	Eukaryotic cell lines
☒	☐	Palaeontology and archaeology
☒	☐	Animals and other organisms
☒	☐	Human research participants
☒	☐	Clinical data
☒	☐	Dual use research of concern

Methods

n/a	Involved in the study	
☒	☐	ChIP-seq
☒	☐	Flow cytometry
☒	☐	MRI-based neuroimaging

Eukaryotic cell lines

Policy information about cell lines

Cell line source(s)	Vero E6 cells: ATCC CCL-81; HEK293T: ATCC CRL-3216; Huh7 were a gift from Dr. Eva Harris (UC Berkeley)
Authentication	Cells were not authenticated.
Mycoplasma contamination	Cells were not tested for mycoplasma.
Commonly misidentified lines (See ICLAC register)	No cell lines in this study are in the ICLAC register.
